# Expression of the *nos* gene and firefly flashing: A test of the nitric-oxide-mediated flash control model

**DOI:** 10.1093/jis/14.1.56

**Published:** 2014-01-01

**Authors:** Hajime Ohtsuki, Jun Yokoyama, Nobuyoshi Ohba, Yoshihiro Ohmiya, Masakado Kawata

**Affiliations:** 1 Division of Ecology and Evolutionary Biology, Graduate School of Life Sciences, Tohoku University, Sendai, Miyagi, Japan; 2 Department of Biology, Faculty of Science, Yamagata University, Yamagata, Yamagata, Japan; 3 Ohba Firefly Research Laboratory, Yokosuka, Kanagawa, Japan; 4 Research Institute for Cell Engineering, National Institute of Advanced Industrial Science and Technology, Osaka, Japan

**Keywords:** bioluminescence, gene expression, Lampyridae, nitric oxide synthase

## Abstract

Fireflies (Coleoptera: Lampyridae) emit various types of light that differ among species and populations of the same species. Their lights are assumed to be biological properties that play important ecological and evolutionary roles. Some species in the Lampyridae emit periodic luminescence, the patterns of which are characterized by speciesspecific intervals. In previous work, it was predicted that the nitric oxide (NO) regulates the oxygen supply required for the bioluminescence reaction of fireflies. Here, the expression of the NO synthase (NOS) mRNA in some fireflies was examined to verify the predictive model of nitric-oxide-mediated flash control in these insects. The expression of the *nos* gene in the lantern organ was observed not only in nocturnal flashing species but also in diurnal non-flashing species. It was shown that the expression levels of *nos* were higher in the lantern of *Luciola cruciata* (Motschulsky) larvae, which that emits continuous light, than in other body parts, although expression in the lantern of the adults, who flash periodically, was not high. Furthermore, there was no significant difference in expression levels among adults of *Luciola cruciata* characterized by different flashing intervals. The data do not support the model of an NO-mediated flash control mechanism, during which oxygen becomes available for the luciferin-luciferase reaction through NO-mediated inhibition of mitochondrial respiration. It is also indicated that flash patterns do not co-vary with NOS production. However, high *nos* expression in the larval lantern suggests that NO may play a role in producing continuous light by functioning as a neurotransmitter signal for bioluminescence.

## Introduction

Bioluminescence is a feature of many species in various taxonomic groups and is used for a variety of purposes, e.g., sexual communication, species recognition, anti-predator defense, and food location ( Lewis and Cratsley 2008 ; Widder 2010 ). Fireflies (Coleoptera: Lampyridae) in particular are well-known and extensively-studied luminous organisms. There are approximately 2000 firefly species throughout the world ( Lawrence 1982 ; Lawrence and Newton 1995) and they emit various types of light (i.e., different flash signal patterns, colors, intensities, etc.) that differ among species ( Lloyd 1971 , 1983; Ohba 2004 ) and even populations of the same species ( Ohba et al. 2001 , Suzuki et al. 2004 ). For instance, among Japanese firefly species, there are several different types of signal patterns, namely, single short pulse, single pulse, single long pulse, continuous light, continuous weak light, and non-luminescent ( Ohba 2004 ). Some species in the Lampyridae emit periodic luminescence, the patterns of which are characterized by speciesspecific intervals. For example, the flash intervals of *Luciola cruciata* (Motschulsky) (Coleoptera: Lampyridae) and *Luciola lateralis* are approximately 2–4 sec ( Ohba et al. 2001 ) and 0.5–1 sec ( Ohba 2001 ), respectively. Intra specific geographic variation in flash intervals may also exist among local populations. In Japan, populations of *L. cruciata* with fast-flash (2-sec type) and slow-flash intervals (4-sec type) are distributed in the western and the eastern regions, respectively.

Although the chemical reactions underlying firefly luminescence are well understood, the mechanism responsible for flashing (the switching on and off of light) remains unknown. It has been suggested that molecular oxygen (O2) is an important factor in the control of the flashing ( Buck 1948 ; Ghiradella 1998 ; Timmins et al. 2001 ; Ghiradella and Schmidt 2004 ). The bioluminescence of fireflies is attributable to the luciferin-luciferase reaction, which involves (1) the production of a luciferin-luciferase-AMP complex and (2) the oxygenation of luciferin, as shown in the following two reactions:

ATP + luciferase + luciferin → luciferase- luciferin-AMP + PPi (Reaction 1)

Luciferase-luciferin-AMP + O2 → luciferase + oxyluciferin + CO2 + AMP + light (Reaction 2).

Luciferin-AMP is formed from luciferin and ATP by the catalytic activity of luciferase in the first reaction, after which luciferase catalyzes the oxidization of luciferin-AMP to energized oxyluciferin via peroxide anion and dioxetane. Finally, energy is released from oxyluciferin, and bioluminescence is emitted. When the light-emitting reaction is initiated *in vitro* by mixing luciferin, luciferase, ATP, and oxygen (Reaction 1 followed by Reaction 2), the maximal light intensity is not emitted until 300 ms after mixing ( DeLuca and McElroy 1974 ). However, maximal light emission occurs within 60 ms when the pre-formed luciferase-luciferin-AMP complex is mixed with oxygen (Reaction 2). In the firefly lantern organ, maximal light is emitted within 100–150 ms after action potential initiation in the brain ( Buck et al. 1963 ). Thus, it has been predicted that firefly flashing is controlled by O2 required for Reaction 2, because Reaction 1 proceeds too slowly to control flashing. Consistent with this hypothesis, some studies have described flash control involving O2 supply mechanisms ( Alexander 1943 ; Buck 1948 ; Wilson and Hastings 1998 ). Kanda (1935) reviewed studies about lantern anatomy and morphology and discussed differences between lanterns of adults and larvae, including Japanese fireflies. The review showed that lanterns in adults of flashing species are more developed and more suitable for O2 regulation in bioluminescence reaction than lanterns of their larval stage. Ghiradella (1977) also showed the anatomical differences of the tracheolar systems among adult and larval lantern in *Photuris* firefly. The adult *Photuris* has welldeveloped structures, including tracheal end cells, which surrounded tracheolar cells, in the lantern that its larva lacks. It is possible that these differences are related to the flash control mechanism involving O2 supply.

Trimmer et al. (2001) and Aprille et al. (2004) have shown that nitric oxide (NO) plays a role in the temporal control of firefly flashing. When NO gas was introduced into a chamber containing North American fireflies ( *Photuris* sp.), flashing began immediately ( Trimmer et al. 2001 ). It has also been observed that octopamine, a neurotransmitter, evokes light production in dissected lanterns, but that light production is inhibited by carboxy-PTIO, an NO scavenger. Trimmer et al. (2001) have also shown that NO synthase (NOS) exists in the firefly lantern in the vicinity of the photocytes. Neurons that innervate the lantern do not terminate directly on the photocytes themselves but synapse on tracheolar cells that surround the terminal branch points of the tracheal air supply ( Smith 1963 ). Thus, it is expected that a mechanism linking photocytes and neurons exists in the lantern. Trimmer et al. (2001) focused on the small free radical gas NO as one potential transmitter that can penetrate cell membranes and quickly cross such distances. They considered that the entry of NO into firefly lanterns leads to a high oxygen concentration by inhibiting the oxygen consumption of mitochondria, and that this triggers the bioluminescence reaction. There after, the light itself releases the inhibition of mitochondrial respiration, and NO is in turn degraded by the resultant high oxygen concentrations. These negative feedbacks result in the light being switched off. Therefore, it is likely that NO is the key determinant in the light-emitting reaction. Although the control of light flashing cannot be explained solely in terms of neuronal signals, such signals do play a role in triggering NO production and light emission. Light flashing may be attributable to oxygen generated by an NO-mediated system and subsequent NO degradation. The NO- mediated model is supported by existing data; however, other mechanisms have been suggested ( Timmins et al. 2001 ; Ghiradella and Schmidt 2004 ), and the model should be further tested. Scientists should consider that NOS is an important factor for bioluminescence, and it is different from other enzymes. NO is predicted to be the factor controlling the tracheal supply of molecular oxygen by inhibiting mitochondrial oxygen consumption.

The purpose of this study was to test the model of NO-mediated flashing control described by Trimmer et al. (2001) . The model predicts that NO production could be lacking in larvae and adult fireflies of non-flashing species that only glow ( Lewis and Cratsley 2008 ). In some firefly species, larvae and adults emit continuous light, and, according to the model, the mechanism of NO releasing oxygen for luciferin oxygenation through inhibition of mitochondrial respiration would require continuous mitochondrial inhibition. Thus, in this case, NO might not be used for flashing (the switching on and off of light); however, NO has an important role for continuous light emission. To test these predictions, we examined *nos* gene expression in various body parts (head, thorax, abdomen, and lantern) of two luminous species of firefly, *Luciola cruciata* and *Luciola lateralis* , and one non-luminous species, *Lucidina biplagiata* (Motschulsky) *. Lucidina biplagiata* is a diurnal species that emits very weak light. In *L. cruciata* , we compared *nos* gene expression levels across different body parts in adults that flash periodically and larvae that emit continuous light. In addition, the relationship between *nos* expression levels and flash patterns at different times and in different populations were also examined. Although there has not been any clear theory explaining the correlation between *nos* expression, NOS production, and flash patterns, we might be able to find the relation if *nos* expression levels are compared among different times and populations in the lanterns of active individuals. To investigate the potential effect of *nos* expression levels on the generation of flash patterns, *nos* gene expression in the lanterns of active (i.e., flying males that flash as part of their courtship behavior) and inactive individuals were examined. To confirm relationships between *nos* expression levels and flash patterns, we also compared expression levels in the lanterns of adult *L. cruciata* collected from different populations (4 sec and 2 sec types) at different times. If the flash patterns correspond to the amount of NO, there can be differences in *nos* expression levels among different flash patterns. Further, it is possible that *nos* expression levels are different between the time that they flash actively for their courtship behavior and other times that they are less active.

## Materials and Methods

### *Luciola lateralis* and *Lucidina biplagiata* collection

Adult males of *L. lateralis* and *L. biplagiata* were collected from Aga (Niigata Pref., Japan) and Sendai (Miyagi Pref., Japan). All individuals were placed separately into 6-cm diameter Petri dishes. The fireflies were reared at 20°C under the same natural light and dark cycle for over 24 hours and were thereafter fixed by freezing in liquid nitrogen at 10:00 or 20:00 local time. Fixed individuals were stored at –80°C until used for total RNA extraction.

### *Luciola cruciata* collection

Adult males of *L. cruciata* were collected from five wild populations, Aomori, Miyagi, Shiga, Okayama, and Kouchi. The populations from Aomori and Miyagi are of the 4 sec (slow) type, whereas those from Shiga, Okayama, and Kouchi are of the 2 sec (fast) type (field observations, data not shown). Resting males were fixed by freezing in dry ice at 17:00, 20:00, 23:00, 2:00, or 5:00 local time in Japan. It is known that the flashing behavior of flying males peaks at around 20:00 ( Ohba 2001 ; Oba and Kainuma 2009 ); therefore, flashing males in flight were captured and were subsequently fixed at 20:00. Collected individuals were stored in ethanol at 80°C until used for total RNA extraction.

### Larvae of *L. cruciata* and *L. lateralis* collection

The larval *L. cruciata* used in this study originated from Chiba Pref., Japan (purchased from Inc. River Fashion, http://ffland.cure.to ). Larval *L. lateralis* were reared at Iwakiri Elementary School, Sendai, Japan. Their sex was not determined. All individuals were placed separately into 6-cm diameter Petri dishes containing water. The larvae were reared at 20°C under the same natural light and dark conditions for over 24 hours and were thereafter fixed by freezing in liquid nitrogen at 10:00 or 20:00 local time. Fixed individuals were stored at –80°C until used for total RNA extraction.

### Total RNA extraction and cDNA synthesis

Adult individuals were separated into four body parts: head, thorax (only the prothorax was used), abdomen, and lantern. Because the lantern of *L. biplagiata* is very small and too difficult to separate, the last two segments of the abdomen containing the lantern were considered as the lantern in this species. Larval individuals were separated into three parts: head and thorax (it was too difficult to separate the head from the thorax), abdomen, and lantern (the segments containing the lantern part). Because segments containing the lantern were treated as the lantern in this study, *nos* expression in the lantern could not be measured exclusively and accurately. However, any difference among segments, including the lantern and other parts in the abdomen, would be reflected as changes in *nos* expression.

Total RNA was isolated from all individuals using RNAiso (TaKaRa, www.takarabio.co.jp ). Contaminating DNA was removed by treating with DNase I (RNasefree; TaKaRa). The RNA concentration was measured using a GeneQuant 100 spectrophotometer (GE Healthcare Biosciences, www.gelifesciences.co.jp ). cDNA was subsequently generated using a PrimeScript® RT reagent kit (Invitrogen, www.invitrogen.com ) in a 10-µL mixture containing 2 µL of 5× PrimeScript® Buffer, 0.5 µL of PrimeScript® RT Enzyme Mix I, 2.5 µM of oligo dT primer, and 5.0 µM of random 6mers. Within the mixture, the concentration of *L. cruciata* RNA was adjusted to 50 ng/µL, and that of *L. lateralis* and *L. bipla-giata* RNA was adjusted to 5 ng/µL.

### Amplification of *L. lateralis* and *L. biplagiata nos*

PCRs for amplification of the *nos* genes of *L. lateralis* (GenBank ID: AB304919; Ohtsuki et al. 2008 ) and *L. biplagiata* (GenBank ID: AB623216; Ohtsuki et al. unpublished) were performed in 10-µL mixtures containing 0.25 units of TaKaRa Ex TaqTM, 1.0 µL of 10× Ex **Table 1 .** Primers used for amplification of *nos* and *rp49* . TaqTM Buffer, 0.2 mM of dNTPs, 0.4 mM of each primer, and 1.0 µL of diluted (10-fold) cDNA solution. The primers used for the amplifications are shown in Table 1 . The following thermal cycle was used: pre-heating at 95°C for 10 sec, followed by 20, 30, 40, or 50 cycles of 95°C for 5 sec and 60°C for 20 sec. The products were analyzed by using 1.2% agarose gel electrophoresis. All PCRs were performed using a Veriti® 200 thermal cycler (Applied Biosystems, www.appliedbiosystems.com ).

**Table 1.**
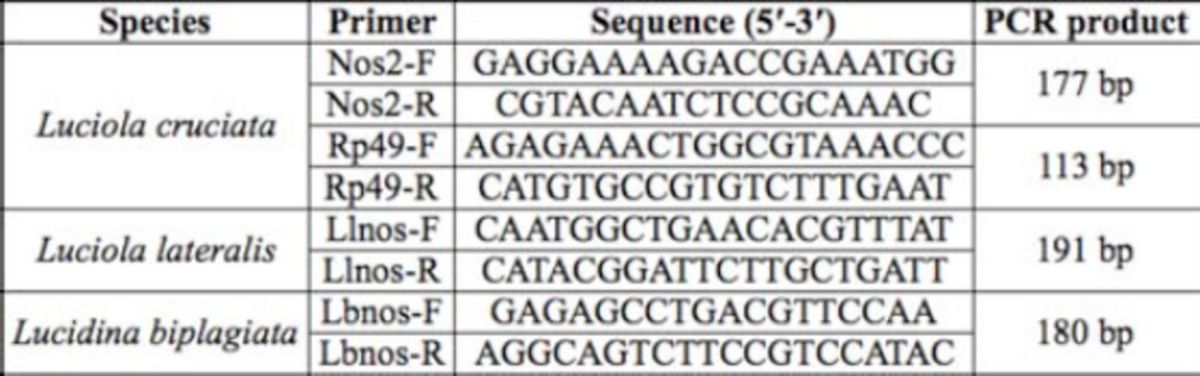
Primers used for amplification of *nos* and *rp49* .

### Quantification analysis of *L. cruciata nos*

Realtime PCR was performed on 20-µL mixtures containing 10 µL of SYBR® Premix Ex TaqTM (TaKaRa), 0.4 mM of each primer, and 2.0 µL of 20-fold diluted cDNA using a LightCycler® 350S system (Roche Diagnostics, www.roche-diagnostics.com ). The thermal cycle conditions for amplification of the *nos* gene of *L. cruciata* (GenBank ID: AB304920; Ohtsuki et al. 2008 ) were as follows: 95°C for 10 sec, followed by 45 cycles of 95°C for 5 sec and 62°C for 20 sec. The primers used for the amplification of *nos* were Nos2-F and Nos2-R ( Table 1 ). The *rp49* gene of *L. cruciata* (GenBank ID: AB205198; Oba et al. 2006 ) was used as an internal control to standardize the results. The thermal cycle conditions for *rp49* were as follows: 95°C for 10 sec, followed by 40 cycles of 95°C for 5 sec and 60°C for 20 sec. The primers used for *rp49* amplification were Rp49-F and Rp49-R ( Table 1 ). The quantitative analysis was performed automatically by the second derivative maximum method using LightCycler® software. The expression levels of *nos* were determined relative to the expression level of *rp49* and were measured three times for each individual.

### Statistical analysis

Statistical analyses were performed using R version 2.12.0 (The R Project for Statistical Computing, www.r-project.org ). Analysis of covariance (ANCOVA) was used to explain the effect of body parts, population, time, and interactions on NOS expression in *L. cruciata* adults and larvae. The differences in the intercept among each regression lines can be detected by ANCOVA assuming that their slopes are the same. Multiple comparisons were performed by Tukey’s HSD post hoc test.

## Results

### Amplification of *L. lateralis* and *L. biplagiata nos*

**Figure 1.**
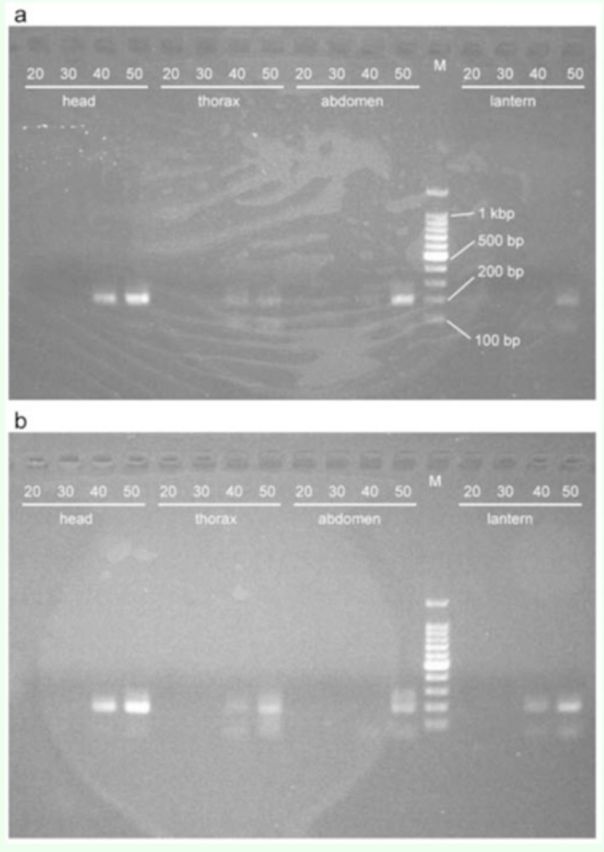
PCR products of *nos* cDNA from adult males of *Luciola lateralis* . The length of the product is 191 bp. The number on each lane indicates the number of PCR cycles. M is the molecular size marker. Individuals were fixed at 10:00 (a) or 20:00 (b) local time. High quality figures are available online.

**Figure 2.**
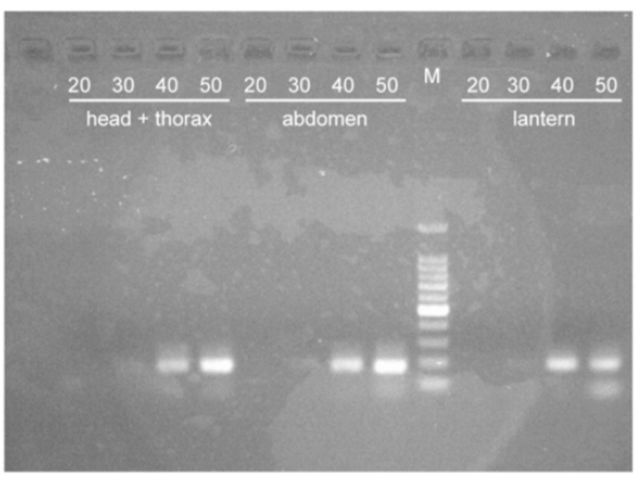
PCR products of *nos* cDNA from larvae of *Luciola lateralis* at 20:00. The length of the product is 191 bp. The number on each lane indicates the number of PCR cycles. M is the molecular size marker. High quality figures are available online.

**Figure 3.**
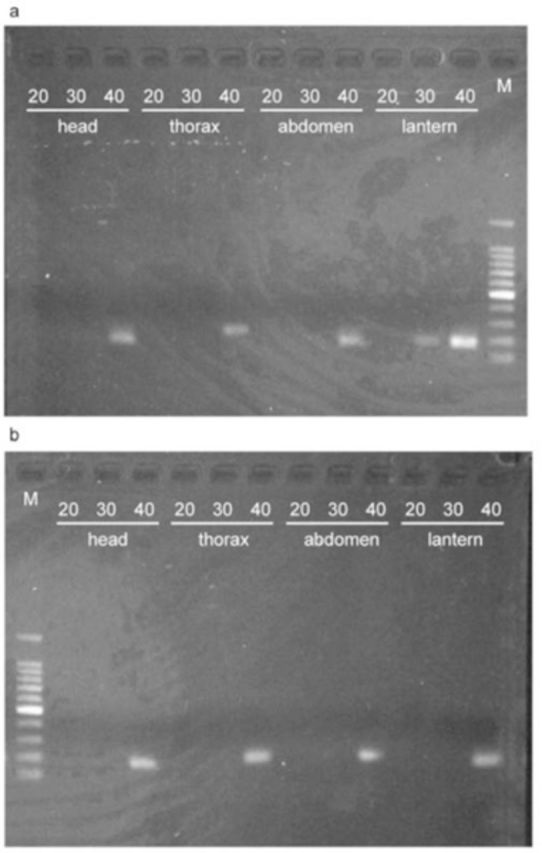
PCR products of *nos* cDNA from adult males of *Lucidina biplagiata* . The length of the product is 180 bp. The number on each lane indicates the number of PCR cycles. M is the molecular size marker. Individuals were fixed at 10:00 (a) or 20:00 (b) local time. High quality figures are available online.

The expected sizes of the PCR products of *nos* from *L. lateralis* and *L. biplagiata* cDNA were 191 bp and 180 bp, respectively. Fragments of approximately 200 bp in both species were confirmed by agarose gel electrophoresis ( Figures 1–3 ). In *L. lateralis* fixed at 10:00, the fragments appeared in the products from the head and thorax after 40- and 50-cycle reactions ( Figure 1a ). In the abdomen and lantern, the fragments appeared in the products of 50-cycle reactions. At 20:00, the fragments appeared in the products from the head, thorax, and lantern after 40- and 50- cycle reactions, whereas for the abdomen the fragment appeared in the product from the abdomen obtained after a 50-cycle reaction ( Figure 1b ). In *L. biplagiata* , the fragment appeared in the lantern at 10:00 after 30- and 40- cycle reactions ( Figure 3a ). Fragments appeared in the products for other body parts and for both time points after 40-cycle reactions (Figures 3a and b). For the larvae of *L. lateralis* , fragments appeared in the products from all body parts after 30- and 40-cycle reactions ( Figure 2 ).

### Expression levels of *nos* in *L. cruciata* larvae

**Figure 4.**
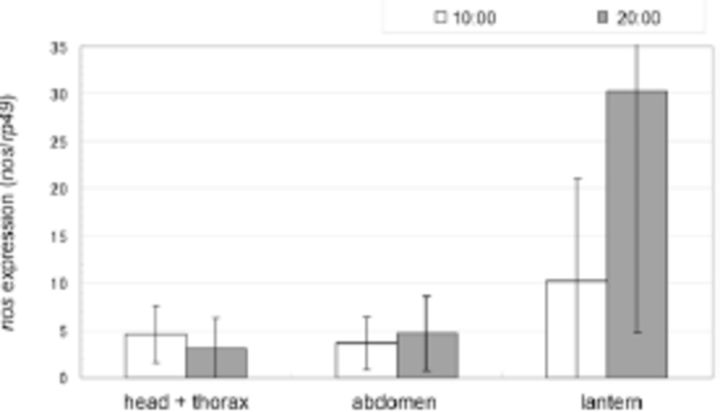
Expression levels of *nos* in larvae of *Luciola cruciata* . Individuals were fixed at 10:00 or 20:00 local time. All data are the average of four individuals. Error bars indicate standard deviation (S.D.). High quality figures are available online.

The expression levels of *L. cruciata nos* in different larval body parts at 10:00 and 20:00 are shown in Figure 4 . There was a significant difference in expression level among body parts (ANCOVA: F (2, 20) = 4.7717, *P* = 0.0202). Expression levels in the lantern were significantly higher than those in the other parts (Tukey’s HSD test: lantern vs. head + thorax, *P* = 0.0424; lantern vs. abdomen, *P* = 0.0392). There was no significant difference between the fixed times (ANCOVA: F (1, 20) = 1.9139, *P* = 0.1818).

### Expression of *nos* in adult *L. cruciata*

**Figure 5.**
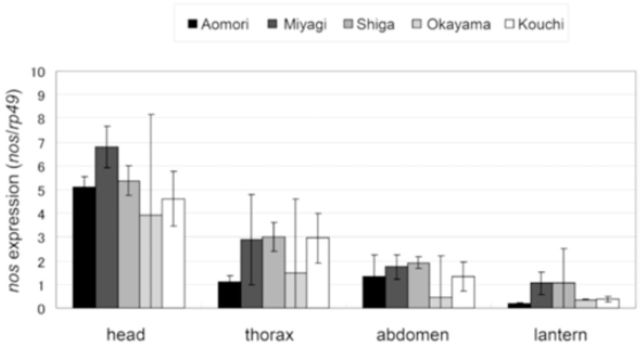
Expression levels of *nos* in flying males of *Luciola cruciata.* Individuals were fixed at 20:00 local time. Those from the Aomori and Miyagi populations are of a slow-flash type (4-sec type), whereas those from the Shiga, Okayama, and Kouchi populations are of a fast-flash type (2-sec type). All data are the average of four individuals. Error bars indicate S.D. High quality figures are available online.

In *L. cruciata* , *nos* expression levels in different body parts of flying males at 20:00 are shown in Figure 5 . We found significant differences in *nos* expression levels among body parts (ANCOVA: F (3, 72) = 40.7960, *P* < 0.0001). The *nos* expression levels in the head were significantly higher than those in the thorax, abdomen, and lantern (Tukey’s HSD test: head vs. thorax, *P* < 0.0001; head vs. abdomen, *P* < 0.0001; head vs. lantern, *P* < 0.0001). Expression levels in the lantern were lower than those in the other parts. We also found significant differences in *nos* expression levels in the lantern and thorax (Tukey’s HSD test: *P* < 0.0001). Although *nos* expression levels in the lantern were slightly lower than those in the abdomen, the difference was not significant (Tukey’s HSD test: *P* = 0.3972). The differences in *nos* expression levels among populations were significant (ANCOVA: F (4, 72) = 3.3666, *P* = 0.01392), but significant differences were not detected after the post hoc test (Tukey’s HSD test: *P* > 0.05).

**Figure 6.**
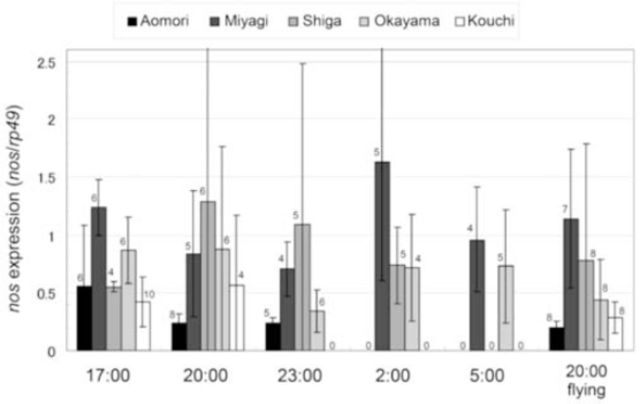
Expression levels of *nos* in the lanterns of resting and flying males of *Luciola cruciata* . Resting individuals were fixed at 17:00, 20:00, 23:00, 02:00, or 05:00 local time. Flying individuals were fixed at 20:00. All data are the average of individuals selected at the same time. The numbers of collected individuals were showed above the bars. Error bars indicate S.D. High quality figures are available online.

The expression levels of *L. cruciata nos* in the lantern of resting males fixed at different times are shown in Figure 6 . There was a significant difference in *nos* expression among populations (ANCOVA: F (4, 98) = 3.5922, *P* = 0.0089). The *nos* expression in the Aomori population (4-sec type) was lower than that in the Miyagi (4-sec type) and Shiga (2-sec type) populations (Tukey’s HSD test: Aomori vs. Miyagi, *P* = 0.0044; Aomori vs. Shiga, *P* = 0.0462). The difference in expression levels was not significant among the fixed times (ANCOVA: F (1, 98) = 3.3627, *P* = 0.0697). At 20:00, there were not significant differences among populations (ANCOVA: F (1, 43) = 0.2812, *P* = 0.1018) and among flying and resting individuals (ANCOVA: F (4, 43) = 2.0671, *P* = 0.5986). However, the expression level in the Miyagi population was higher than that in the Aomori population at 20:00 (Tukey’s HSD test: *P* = 0.0493).

## Discussion

The results of the present study do not appear to support the model of the NO-mediated flash control mechanism described by Trimmer et al. (2001) . In this study, we concentrated on the expression of NOS mRNA, and it does not always reflect abundance and activity of NOS protein. Therefore, it does not mean that the higher expression of NOS mRNA indicates the presence of a larger amount of NO. However, our results would seem to be contrary to the NO-mediated model. According to the model, NO production, triggered by neural stimulation, causes an increase in O2 concentration for the luciferin-luciferase reaction, which in turn initiates light emission. Subsequently, negative feedback due to NO degradation causes light to be switched off ( Trimmer et al. 2001 ). Our results show that NOS mRNA is expressed not only in the lantern of the nocturnal, flashing species *L. cruciata* and *L. lateralis* , but also in *L. biplagiata* , a diurnal, non-flashing species. Further, *nos* expression was higher in the lantern of *L. cruciata* larvae than in other body parts, although expression in the lantern of adults was low. Adult *L. biplagiata* are diurnal and non- luminescent, but sometimes emit very weak continuous light ( Ohba 1983 ). Larval *L. cruciata* do not flash periodically, but emit continuous weak light. NO production in larvae of flashing and an adult of non-flashing species indicates that NO does not mediate flashing by inhibiting mitochondria, thus resulting in higher oxygen availability in the lanterns of fireflies.

However, our results do not necessarily disprove that bioluminescence is induced by the function of NO function as a neuronal messenger. NO is an important molecule in the nervous system of insects, and it plays an important role in the NO/cyclic guanosine monophosphate (cGMP) signaling mechanism ( Davies 2000 ; Bicker 2001 ). In neurons, neuronal activity leads to Ca2+ influx, which stimulates NOS. NOS catalyzes the production of NO and L-citrulline from L-arginine, O2, and NADPH-derived electrons. NO rapidly diffuses and reaches target cells, thus acting as a neuronal messenger. NO binds to a heme moiety in soluble guanylyl cyclase, resulting in the stimulation of the enzyme and consequent elevation of cGMP concentration in the target cell. The resulting increase in intracellular cGMP levels has multiple effects, such as activation of ion channels, cGMP-dependent kinases, and cGMP-dependent phosphodiesterases. Thus, there might be a possibility that NO functions to induce rapid pulses of bioluminescence.

Our results showed that the expression levels of *nos* in the lantern of larval individuals were very high. This suggests that a large amount of NO is used in this part of the body. The lantern of larvae is smaller than that of adults, and larvae do not flash periodically. One possibility is that NOS is used not for light emission, but for some other purpose in the region near the lantern. In other insects NOS activity or *nos* gene expression is observed in the midgut, fat bodies, and Malpighian tubules ( Davies 2000 ; Hao et al. 2003 ; Faraldo et al. 2007 ). In the land crab *Gecarcinus lateralis* , *nos* is expressed in the Y-organ, which is associated with the regulation of molting ( Kim et al. 2004 ). However, in the separated body parts, including the lantern, used for RNA extraction, no organ consuming large amounts of NO was identified.

High levels of NOS in larval lanterns, together with the observed effects on *nos* expression in lanterns of adult fireflies of *L. biplagiata,* suggest that NO may also play a role in producing lengthy, weak bioluminescence. Larvae emit light as a defense mechanism against predators or to attract prey ( Costa and Vanin 2010 ). A diurnal species, *L. biplagiata,* also emits very weak (or almost no) light, suggesting similar roles for bioluminescence as those in larvae. For both larvae and diurnal species, NO might function as a neurotransmitter for continuous weak light, which may be related to low O2 conditions. It has been shown that *Photuris pennsylvanica* exhibits periodic flashing in normal air conditions but emits continuous weak light under conditions of low O2 tension ( Snell 1932 ). This suggests that the mechanism regulating flash interval is not operative when O2 supply is insufficient. It is possible that low O2 levels are responsible for the continuous weak light observed in larvae.

Adult fireflies have welldeveloped lanterns, which regulate O2 concentrations for the bioluminescence reaction (Dahlgren 1917; Peterson and Buck 1968 ). The lantern of larval fireflies is undeveloped, and the tracheoles within the larval lantern are not extensively branched ( Oertel et al. 1975 ; Ghiradella 1977 ). The efficient control of O2 supply for bioluminescence reaction would be difficult in the larval lantern because it does not have such the specialized tracheal system that would be required for the flash control in its adult stage. It might be cause for a greater loss of O2. Therefore, it is possible that a larger amount of NO is required to gain sufficient O2 for light emission in larvae than adults. Moreover, in *L. cruciata* , adults flash while flying at night. Although larvae also emit light at night, they live underwater and are consequently exposed to lower concentrations of dissolved oxygen. It is also likely that a large amount of NO is required to generate O2 in water for light emission in larvae of aquatic species such as *L. cruciata* . As above, it is relatively difficult for larvae to obtain sufficient O2 for light emission relative to adults because of differences in lantern structure and habitat. And, a large amount of NO would be correlated to continuous light in larvae or others that fail to control their flash patterns. Continuous light could require larger amounts of NO than a short-duration flash because NO may rapidly degrade once it produces bioluminescence. High levels of *nos* expression, and probably a large amount of NO generated by NOS, in larval lanterns would be consistent with the need for maximum signal to emit light under conditions of low O2.

In flying individuals of *L. cruciata* , it was found that *nos* expression in the head was higher than in other body parts. It is known that NO is involved in memory formation, vision, and olfaction in the head of insects ( Müller 1997 ; Davies 2000 ). In the case of fireflies, we assume that expression of the *nos* gene observed in the head is associated with the functioning of their compound eyes. The compound eyes of nocturnal species of Lampyridae are larger than those of diurnal species ( Ohba 2004 ). *Luciola cruciata* , in particular, needs to distinguish the flash intervals of other individuals because they have mating preferences for individuals with specific flash intervals. It is possible that expression of the *nos* gene in compound eyes is related to the recognition of flash intervals in the dark of the night.

Our results showed that there was no difference in *nos* expression levels of *L. cruciata* across fixed times and that *nos* expression in *L. cruciata* was not higher in the lanterns of flashing males captured in flight at 20:00— when flashing behavior peaks—than at other times. In addition, there was no significant difference in *nos* expression among populations of 2-sec (fast) and 4-sec (slow) types. However, there were some significant differences in the expression levels among different populations. Expression levels in the Aomori population (4-sec type) were lower than those in the Miyagi (4-sec type) and Shiga (2-sec type) populations, irrespective of flash interval. These results indicate that there may not be a clear correlation between *nos* expression and flash patterns. To date, there has been no clear explanation as to how NO, acting as a neural signal, can affect flash patterns. In addition, how NO production is related to *nos* expression in the lantern of fireflies is unknown. Thus, our negative findings regarding the relationship between *nos* expression levels and flashing patterns should not be used as evidence for immediate rejection of the hypotheses that NO production is related to different flashing activities and that NO acts as a neurotransmitter signal for bioluminescence. There might be other mechanisms that generate the various flash patterns. The role of octopamine, a neurotransmitter that evokes the light-emitting reaction should be investigated; alternatively, other unknown factors that play a role in the neural generation of flash signaling patterns should be determined.

## Abbreviations

NO: nitric oxide
NOS: nitric oxide synthase
